# Neuronal Zinc Transporter ZnT3 Modulates Cerebral Ischemia-Induced Blood-Brain Barrier Disruption

**DOI:** 10.14336/AD.2023.1011

**Published:** 2023-10-18

**Authors:** Zhifeng Qi, Xixi Zhou, Wen Dong, Graham S. Timmins, Rong Pan, Wenjuan Shi, Shuhua Yuan, Yongmei Zhao, Xunming Ji, Ke Jian Liu

**Affiliations:** ^1^Department of Neurology, Cerebrovascular Diseases Research Institute, Xuanwu Hospital of Capital Medical University, Beijing, China.; ^2^Department of Pharmaceutical Sciences, University of New Mexico Health Sciences Center, Albuquerque, NM 87131, USA.; ^3^Department of Pathology, Stony Brook University, Stony Brook, NY 11794, USA

**Keywords:** zinc, blood-brain barrier, cerebral ischemia, matrix metalloproteinase-2 (MMP-2), zinc transporter 3 (ZnT3)

## Abstract

Zinc plays important roles in both physiological and pathological processes in the brain. Accumulation of free zinc in ischemic tissue is recognized to contribute to blood-brain barrier (BBB) disruption following cerebral ischemia, but little is known either about the source of free zinc in microvessels or the mechanism by which free zinc mediates ischemia-induced BBB damage. We utilized cellular and animal models of ischemic stroke to determine the source of high levels of free zinc and the mechanism of free zinc-mediated BBB damage after ischemia. We report that cerebral ischemia elevated the level of extracellular fluid (ECF-Zn) of ischemic brain, leading to exacerbated BBB damage in a rat stroke model. Specifically suppressing zinc release from neurons, utilizing neuronal-specific zinc transporter 3 (ZnT3) knockout mice, markedly reduced ECF-Zn and BBB permeability after ischemia. Intriguingly, the activity of zinc-dependent metalloproteinase-2 (MMP-2) was modulated by ECF-Zn levels. Elevated ECF-Zn during ischemia directly bound to MMP-2 in extracellular fluid, increased its zinc content and augmented MMP-2 activity, leading to the degradation of tight junction protein in cerebral microvessels and BBB disruption. These findings suggest the role of neuronal ZnT3 in modulating ischemia-induced BBB disruption and reveal a novel mechanism of MMP-2 activation in BBB disruption after stroke, demonstrating ZnT3 as an effective target for stroke treatment.

## INTRODUCTION

Zinc, as a richly distributed metal ion in the central nervous system, is essential in neuronal development, learning and memory [1, 2]. Despite this essentiality, numerous studies have demonstrated that excessive free zinc (Zn^2+^, defined as loosely bound zinc, that is histochemically detectable and removable by chelating agents) contributes to cerebral ischemic injury [3, 4], indicating its homeostasis is also essential. Our recent studies showed that ischemia/reperfusion induces free zinc accumulation in cerebral microvessels and causes blood-brain barrier (BBB) disruption [5, 6], which plays a crucial role in secondary inflammation and hemorrhagic transformation. However, neither the source of elevated free zinc levels in microvessels nor the mechanism(s) by which it causes BBB disruption are known.

It is known that large amounts of zinc exist in neurons, particularly in synaptic vesicles [7]. The high concentrations of free zinc inside neurons under ischemic conditions results in neuronal death through upregulating the generation of reactive oxygen species (ROS) and glutamate toxicity [8, 9]. At the same time, free zinc may also be released into the extracellular fluid (ECF-Zn) during the process of cerebral ischemia and reperfusion [1, 10]. Thus far, free zinc release from synaptic vesicles is well documented in response to a variety of stimuli. Zinc transporter 3 (ZnT3), generally expressed in synaptic vesicles, is reported to be responsible for zinc transportation from synaptic vesicles into the synaptic cleft, modulating post-synaptic receptors [11]. However, it is unclear whether ZnT3 is responsible for elevating ECF-Zn levels during cerebral ischemia and whether this process plays a significant role in ischemia-induced BBB damage.

Matrix metalloproteinases (MMPs) constitute a large family of endopeptidases with a zinc-binding site at the catalytic domain. MMP-2/-9 are mainly secreted by resident or inflammatory cells into the extracellular surroundings (ECF-MMPs) to degrade extracellular matrix, such as tight junction proteins between endothelial cells, which leads to BBB disruption after cerebral ischemia [12, 13]. The traditional mechanism of MMP activation has been generally accepted as “cysteine-zinc switch” that a carboxyl terminus cysteine acts as a fourth inactivating ligand for the catalytic zinc atom in the active site and rendering the enzyme inactive [14]. MMP-2/-9 is then activated when this cysteine-zinc pairing is disrupted and the active site is exposed to cleave its substrates, such as tight junction proteins. Our recent studies reported that free zinc accumulation in brain tissue leads to elevated MMP-2/-9 activation during cerebral ischemia [5, 6]. However, nothing is known about whether and how ECF-Zn regulates ECF-MMPs activation, nor the role and mechanisms of ECF-Zn in ischemia-induced BBB damage.

In this study, utilizing cellular and animal models of ischemic stroke, including transgenic mice with ZnT3 specifically knockout in neurons, we determined whether ECF-Zn contributes to BBB damages during cerebral ischemia, and investigated both the source of high levels of ECF-Zn and the mechanism by which ECF-Zn mediates ischemia-induced BBB damage.

## MATERIALS AND METHODS

### Data availability

The data are available from the corresponding author upon reasonable request.

### Animals

All animal experiments were approved by the Institutional Animal Care and Use Committee of Xuanwu Hospital of Capital Medical University (Beijing, China) and performed according to ARRIVE guidelines. Animals were housed in pathogen-free conditions under a standardized light-dark cycle with free access to food and water. Animal surgeries were performed under anesthesia. Group design, surgeries and outcome assessments were performed blindly to investigators. Animals were randomized into groups using a random number table.

Sprague-Dawley (SD) rats were purchased from Beijing Vital River Laboratory Animal Co., Ltd. Transgenic mice with ZnT3 specifically knockout in neurons (Slc30a3^fl/fl; Syn1-icre/+^, ZnT3-cKO) were generated at the Beijing Biocytogen Co. Ltd. The littermates (Slc30a3^fl/fl;+/+^) of ZnT3-cKO mice were used as control.

### Transient focal cerebral ischemia animal model

Transient focal cerebral ischemia was induced in male SD rats (8 week, 290-320g) or transgenic mice (8 week, 22-25g) by middle cerebral artery occlusion (MCAO). Male animals were used in the present study to increase the homogeneity to test our mechanistic hypothesis and to make direct comparisons to literatures where overwhelmingly majority studies were carried out in male rodents.

Animals underwent 120-min MCAO followed by 4h reperfusion using the suture (Cat: 403956PK5RE for rats, 602156PK5Re for mice, Doccol, USA), as we previously described [15]. Animals were anesthetized with 2% isoflurane and underwent 120-min MCAO followed by 4h reperfusion using the suture. We used isoflurane anesthesia only for 2h when collecting dialysate during ischemia. After removing the suture, anesthesia was no longer applied during reperfusion. We considered 70% drop of cerebral blood flow by laser-doppler-flow monitoring as the ischemia criterion and also confirmed when animals appeared circling to the ischemic lateral after MCAO surgery. Animals with subarachnoid hemorrhage or without circling were excluded. Animals in the sham group underwent the same procedure of surgery except for inserting the suture.

### Drug administration in animals

CaEDTA is a membrane-impermeable zinc chelator. In this study, we hypothesized that extracellular zinc contributes to BBB disruption during cerebral ischemia. Therefore, we applied CaEDTA (Cat: ED2SC, Sigma) to remove the extracellular free zinc that was released into the matrix during ischemia. CaEDTA (50nmol in 5μl saline) was microinjected into the lateral ventricle of rats (1mm posterior to bregma, 1.5mm lateral to the midline, and 4mm below the surface of the brain) 30min prior to MCAO surgery, as previously reported [16-18].

To investigate the role of serum zinc in BBB damage, CaEDTA (50μmol/kg) or zinc chloride (80μmol/kg) in 1ml saline was administered intravenously 30min before MCAO surgery to remove the free zinc in serum. The intravenous dose of CaEDTA was based on its application in patients suffering heavy metal poisoning [19]. while the dose of zinc chloride was chosen to mimic the level of zinc in hyperzincemia seen clinically [20]. The doses of drugs were converted according to the body weight ratio between human beings and rodents. The same volume of saline was given as the vehicle control.

For ZnT3-cKO mice, zinc chloride was administered into the lateral ventricle to elevate the concentration of free zinc in extracellular surroundings during cerebral ischemia. Zinc chloride (60nmol in 2μl saline, Cat: Z0152, Sigma-Aldrich) was microinjected into lateral ventricle 30min before MCAO. The same volume of saline was given in the control group.

### Microdialysis and extracellular fluid collection

Microdialysis was performed to collect extracellular fluid samples from the interstitial space of ischemic brain tissue, according to our previous report [21]. Briefly, right after ischemia onset, a microdialysis probe (Cat: 8309664, CMA12; 4mm; 100kDa cutoff membrane, CMA/Microdialysis) was surgically placed into the striatum in ischemic lateral using the coordinates (Rats: 0.3mm posterior to bregma, 4mm lateral to the midline, and 7mm below the surface of the brain; Mice: 1mm anterior to bregma, 1.8mm lateral to the midline, and 2.5mm below the surface of the brain). Then, the inlet and outlet tubing were connected to a microdialysis pump (CMA/Microdialysis). Probes were perfused with saline with the following sequence: 5μL/min for 10min, 2μL/min for 10min, and 2μL/min for 90min until the end of MCAO. Because of the limited amount of CSF collected, we pooled the samples for only two time periods (30-75 and 75-120 min after ischemia onset). The collected extracellular fluid was used for measuring the level of free zinc and MMP-2 activity with zymography. Animals were under anesthesia during sample collection.

### Measurement of BBB disruption after 2h ischemia/4h reperfusion

Previous studies have reported a biphasic opening of the BBB after MCAO, occurring first at 3-5h, then at 48-72h of recirculation [22, 23]. Studies have shown that the early phase of BBB disruption is associated with hemorrhagic transformation, inflammation, and edema after recanalization following acute ischemic stroke. Our previous study showed that free zinc accumulated in ischemic microvessels and led to increased BBB permeability at 4 h reperfusion [5]. Therefore, in the present study we focused on the role of neuronal zinc transporter ZnT3 in modulating cerebral ischemia-induced BBB disruption at 4h reperfusion.

Evan’s Blue (EB) or endogenous IgG leakage was measured to evaluate the BBB disruption, as we previously reported [5]. EB (2%, Cat: 056-04061, WaKo, Japan) was intravenously administered at 2h following reperfusion. At the end of 4h reperfusion, intravascular EB was removed from circulation by transcardial perfusion with saline. The whole brain was collected and cut into coronal slices (2mm-thick for rats; 1mm-thick for mice). Brain slices were photographed and non-ischemic and ischemic hemispheric tissues were weighed and homogenized in trichloroacetic acid (Cat: 76-03-9, Sigma-Aldrich). The supernatant was diluted 4-fold with ethanol, and fluorescence intensity was measured quantitatively on a microplate fluorescence reader. For endogenous IgG leakage experiment, the rats were transcardially perfused with ice-cold PBS at the end of reperfusion. A 3-mm-thick brain region (approximately spanning from 0 to -3.0 mm relative to the bregma) was quickly cut and fixed in 4% paraformaldehyde. A set of 3 consecutive 3-μm-thick slices was generated at 1mm interval. Each slice was stained with FITC-conjugated goat anti-mouse IgG (Cat: ZB-2305, ZSbio, China) and the endogenous IgG leakage was visualized using fluorescence microscope.

### Measurement of cerebral infarction and neurological functions

The cerebral infarction and neurological functions were measured after 2h ischemia plus 22 h reperfusion, as previously reported [5, 24]. Brains tissues were stained by 2% 2,3,5-triphenyltetrazolium chloride (TTC, Cat: T8877, Sigma-Aldrich). The cerebral infarction volume was analyzed using Image J software. Neurological functions were measured using modified neurological severity scores (mNSS) and the foot-fault test following 2h ischemia plus 22 h reperfusion in a double-blind manner.

### Conditional culture medium collection from endothelial cells after oxygen/glucose deprivation exposure

**Figure 1.**
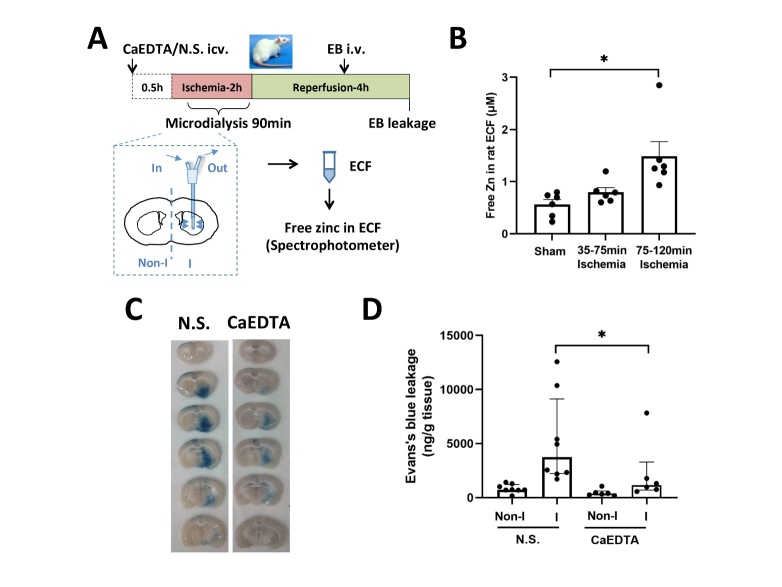
**Elevated level of free zinc in extracellular fluid (ECF-Zn) exacerbates ischemia-induced BBB damage. (A)** The experimental schematic diagram. Extracellular fluid (ECF) of MCAO rats was collected from 30 to 120 min of MCA occlusion to determine the concentration of ECF-Zn in ischemic brain. CaEDTA (a membrane-impermeable zinc chelator, 50nmol in 5μl saline), was microinjected into lateral ventricle to remove ECF-Zn 30min before onset of MCAO. At the end of 4 h reperfusion, Evan’s blue dye (EB) extravasation was measured to evaluate the BBB permeability with or without CaEDTA treatment. icv: lateral ventricle microinjection; iv: intravenous injection. **(B)** ECF samples during the early phase (30-75 min since ischemia onset) or the later phase of ischemia (75-120 min) were separately collected and analyzed for ECF-Zn by spectrophotometry. ECF in Sham group was analyzed as the baseline. * p<0.05, versus Sham group (n=6 in each group). **(C/D)** Evan’s Blue leakage in CaEDTA-treated and normal saline control (N.S.) groups. I: ischemic hemisphere; Non-I: non-ischemic hemisphere. (N.S.: n=8; CaEDTA: n=6). * p<0.05. Data are presented as mean±SEM.

The mouse brain microvascular endothelial cell line (bEnd3) was obtained from American Type Culture Collection (Manassas, VA, USA). These endothelial cells (ECs) were cultured on inserts in Dulbecco’s modified Eagle medium (DMEM, Cat: C11995500BT, Gibco) with 10% FBS (Cat: 10091148, Gibco) and Antibiotic-Antimycotic (Cat: 15070063, Gibco) at a humidified atmosphere of 5% CO_2_/95% air at 37°C. The ECs were prepared for as an in vitro BBB model when the trans-epithelial electrical resistance reached to 300Ω×cm^2^, as we previously reported [5]. To examine the role of free zinc in regulating MMP2 activity, different doses (final concentration 0~1.5μM) of zinc chloride were added to the culture medium of ECs. The doses were chosen according to the concentration of free zinc in the microdialysis samples of extracellular fluid from ischemic animals (Fig. 1B).

Oxygen/glucose deprivation (OGD) was applied to ECs to mimic cerebral ischemia in vivo, as we described previously [5]. In brief, the glucose-free DMEM culture medium (Cat: 11966-025, Gibco) was pre-equilibrated with 95% N_2_/5% CO_2_ for 15min and added to culture ECs. Then ECs were placed in a humidified airtight chamber (Billups-Rothberg Inc., Del Mar, CA, USA) flushed with 95% N_2_/5% CO_2_ for 15min. The chamber was sealed and kept at 37°C for 2h. OGD exposure was terminated by removing plates from the hypoxic chamber. Before returning ECs to the normal incubator (5% CO_2_/95% air), glucose (1mg/ml) and FBS (10%) were supplemented into the cultured medium. After 4-h reoxygenation, the culture medium was collected for MMP-2 zymography.

### Spectrophotometric measurement of free zinc

The level of free zinc in extracellular fluid and serum was quantitatively measured using the 4-(2-pyridylazo) resorcinol (PAR, Cat: 323209, Sigma-Aldrich) colorimetric assay, as previously described [25]. PAR (final 1mM) was added to samples (1:10) and the spectra was scanned at 493nm on a spectrophotometer. The zinc concentration was calculated from the optical density using a standard curve.

### MMP-2 purification by immunoprecipitation

MMP-2 protein was purified by immunoprecipitation from ECs cultured medium, animal microdialysis sample, or brain homogenate (200μg total proteins) using MMP-2 antibody (Cat: 87809, Cell Signaling Technology), according to our previous study [26], and the manufacturer’s instruction of Dynabeads Protein A Immunoprecipitation Kit (Cat: 10006D, Thermo Fisher Scientific). The purified MMP-2 was collected for quantitating zinc binding in purified MMP-2 or measuring MMP-2 activity by gelatin zymography.

### Measurement of free zinc interaction with purified MMP-2

To determine the interaction of free zinc with MMP-2, different concentrations (0~1.5μM) of zinc chloride were incubated at 37°C with purified MMP-2 bonded with beads (see MMP-2 purification by immunoprecipitation above). N,N,N’,N’-Tetrakis (2-pyridylmethyl) ethylenediamine (TPEN, 5μM, Cat: P4413, Sigma-Aldrich), a specific zinc chelator, was used to block the zinc binding to MMP-2. After 2h incubation, the pellet (purified MMP-2 bonded with beads) was collected and rinsed with PBS for measurement of zinc content in MMP-2 protein and the activity of MMP-2.

### Measurement of zinc content in purified MMP-2

The purified MMP-2 was further incubated with 100μl of 0.1M citric acid (pH=3.0, Cat: 251275, Sigma-Aldrich) for 30min, followed by centrifugation at 10,000rpm for 5min at 4°C. The resultant supernatant was adjusted to pH=7 with 10N sodium hydroxide and then incubated with 10mM hydrogen peroxide for 3h at 4°C to release zinc from protein[27]. The amount of released zinc from MMP-2 protein was assayed spectrophotometrically and/or using inductively coupled plasma-mass spectrometry (ICP-MS, PerkinElmer NexION 2000). For ICP-MS, spiked samples and blanks were included with experimental samples as additional quality control for preparation and analyses [28].

### Gelatin zymography assay for MMP-2 activity

Gelatin zymography was performed to evaluate the activity of purified MMP-2 from extracellular fluid, ECs culture medium or brain homogenates, as we described previously[5]. Samples of purified MMP-2 were loaded into 10% SDS-polyacrylamide gels copolymerized with 1mg/mL gelatin. Gels were washed and incubated with a developing Tris-buffer before staining with Coomassie blue R-250 (Cat: B0149, Sigma-Aldrich). Gels were then destained and MMP-2 intensities were quantified.

### Cerebral microvessel isolation

To specifically investigate the impacts of zinc release on occludin degradation in cerebral microvessels, we extracted cerebral microvessels from the brain tissue of rats and mice after ischemia/reperfusion, as we described previously [5]. Briefly, at the end of reperfusion, cerebral microvessels were isolated from the ischemic hemispheres. The brain tissue was dissected and homogenized in ice-cold PBS. The homogenates were filtered through a 41-μm nylon mesh. Microvessels retained on the mesh were purified with Dextran T-500 and stored at -80°C for Western blot.

### Western blotting for measuring occludin in isolated cerebral microvessels or MMP-2 in extracellular fluid

Homogenates (20μg protein) of microvessels, purified MMP-2 from extracellular fluid, or brain tissue of ZnT3-cKO mice were prepared for Western blot, as previous reported [15]. The primary antibodies were: ZnT3 (Cat: AZT-013, 1:1000, Alomone Labs); ZnT1 (Cat: AZT-011, 1:1000, Alomone Labs); MMP-2 (Cat: 87809, 1:1000, Cell Signaling Technology); occludin (Cat: 711500, 1:500, Invitrogen); zonula occludens 1 (ZO-1, Cat: 40-2200, 1:1000, Invitrogen), β-actin (Cat: TA-09, 1:2000, ZSGB-Bio). Quantification of protein levels was expressed as the ratio to β-actin.

### Immunohistochemistry staining

To determine the expression of ZnT3 in brain tissues, the brain slices of ZnT3-cKO or wild type mice were stained using immunohistochemistry staining, as previously reported [15]. The antibodies were as follows: ZnT3 (Cat:AZT-013, 1:250, Alomone Labs, Israel); NeuN (a neuronal nuclear specific marker, Cat: AG5317, Beyotime, CHN), microtubule-associated protein 2 (MAP2, a specific biomarker of axons and dendrites in neurons, Cat: MA5-12826, Thermo Fisher Scientific), glial fibrillary acidic protein (GFAP, a specific biomarker of astrocytes, Cat:12096, Servicebio, CHN) and CD34 (a specific biomarker of microvessels, Cat:14034, Servicebio, CHN). Secondary antibody only controls were used to validate antibody specificity and distinguish genuine target staining from background. Images were captured after 4',6-diamidino-2-phenylindole, dihydrochloride (DAPI, Cat: 62247, Thermo Fisher Scientific) staining.

### Statistical analysis

All data were analyzed using the IBM SPSS Statistics for Windows version 26.0 (IBM Corp, Armonk, NY, USA). Normal distribution was determined using Kolmogorov-Smirnov test. For normal distribution data, the group differences were compared using the two-independent sample-test or one-way analysis of variance with least significant difference (LSD) test. For non-normal distribution data or small samples (n<6), the group differences were compared using the two-independent Mann-Whitney U test or Kruskal-Wallis test with Bonferroni correction. LSD test or Bonferroni correction was employed for adjusting each p value of group differences, and more than 3 tests were performed to compare the p value. A value of *p*<0.05 was considered statistically significant. Plots were generated using GraphPad Prism 9.4.0 (GraphPad Software Inc., San Diego, CA, USA).

## RESULTS

### Elevated level of free zinc in extracellular fluid (ECF-Zn) exacerbates ischemia-induced BBB damage

To investigate the role of extracellular free zinc (ECF-Zn) in BBB damage during ischemia, brain extracellular fluid of rats undergoing middle cerebral artery occlusion (MCAO) was collected using microdialysis for 90 min (30-120 min during MCAO) to determine the concentration of ECF-Zn in ischemic brain. CaEDTA (a membrane-impermeable zinc chelator, 50nmol in 5μl saline) was microinjected into the lateral ventricle to selectively remove ECF-Zn 30 min before onset of MCAO. Animals in the control group followed the same procedure but were injected with saline. At the end of 4h reperfusion, Evan’s blue dye extravasation was measured to evaluate the BBB permeability with or without CaEDTA treatment. The experimental schematic diagram is shown in Figure 1A. In order to investigate the changes of ECF-Zn during cerebral ischemia, ECF was collected separately during the early phase of ischemia (30-75 min after stroke onset) or later phase of ischemia (75-120 min after stroke onset). Free zinc measurement using a spectrophotometric assay (Fig. 1B) showed that the ECF-Zn only slightly increased during the early phase of ischemia (30-75 min), while it had a 3-fold increase during later phase of ischemia (75-120 min) as compared to sham control, indicating that ischemia caused a dramatic elevation of free zinc in the ischemic tissue. The EB leakage data (Fig. 1C) show that the area of Evan’s Blue (EB) extravasation was much smaller in CaEDTA treated rats than those in the saline control group. Quantitative analysis of EB content in brain tissues (Fig. 1D) showed that removing ECF-Zn with CaEDTA greatly reduced EB extravasation, suggesting that ECF-Zn could play an important role in mediating the ischemia-induced BBB damage.

### Altering the concentration of free zinc in serum (Serum-Zn) does not change ECF-Zn level nor BBB permeability during cerebral ischemia

**Figure 2.**
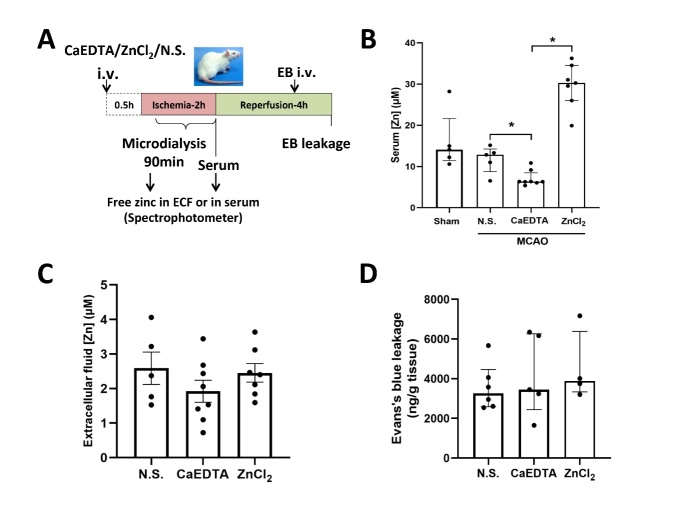
**Altering the level of free zinc in serum (Serum-Zn) does not lead to significant change of ECF-Zn or BBB permeability during cerebral ischemia. (A)** The experimental schematic diagram. CaEDTA, ZnCl_2_ or saline was pre-administered intravenously to alter the level of Serum-Zn in rats 30min before MCAO. The ECF was collected during ischemia for 90min (from 30min to the end of MCAO). Sera were collected at the end of ischemia to analyze the level of Serum-Zn. EB leakage was analyzed to evaluate the BBB permeability at the end of 4-h reperfusion. iv: intravenous injection. **(B)** Spectrophotometric measurement of Serum-Zn following 2-h cerebral ischemia in sham group or in MCAO groups with saline (N.S.), CaEDTA or ZnCl_2_ intravenous injection. (Sham: n=5; MCAO+N.S.: n=5; MCAO+CaEDTA: n=8; MCAO+ZnCl_2_: n=7). *p<0.05. **(C)** Spectrophotometric measurement of ECF-Zn in microdialysates. (MCAO+N.S.: n=5; MCAO+CaEDTA: n=8; MCAO+ZnCl_2_: n=7). **(D)** Quantitative analysis of Evan’s Blue leakage in CaEDTA, ZnCl_2_, or N.S. control group following cerebral ischemia. (MCAO+N.S.: n=6; MCAO+CaEDTA: n=5; MCAO+ZnCl_2_: n=4). Data are presented as mean±SEM.

We investigated the source for the elevated level of ECF-Zn during cerebral ischemia. Studies showed that free zinc in plasma (Serum-Zn) is maintained at concentrations of 12-15μmol/L in healthy adult humans [29]. It is possible that Serum-Zn is a potential source of ECF-Zn by entering brain tissues across the compromised BBB during ischemia. To test this possibility, CaEDTA, ZnCl_2_ or saline was pre-administered intravenously to alter the level of Serum-Zn in rats 30min before MCAO. The ECF was collected from 30 to 120 min of MCAO. Sera were also collected at the end of ischemia to analyze the level of Serum-Zn. EB leakage was analyzed to evaluate the BBB permeability at the end of 4-h reperfusion. The schematic diagram is shown in Figure 2A. There was a slight decrease in the Serum-Zn after 2-h cerebral ischemia. CaEDTA injection intravenously was able to significantly reduce Serum-Zn levels, while ZnCl_2_ pretreatment greatly elevated it (Fig. 2B). However, ECF-Zn remained relatively unchanged by administration of either CaEDTA or ZnCl_2_ (Fig. 2C), even though they greatly changed Serum-Zn levels (Fig. 2B). BBB permeability analysis showed that intravenous administration of CaEDTA or ZnCl_2_ did not affect the BBB damage during cerebral ischemia (Fig. 2D). These results indicate that modulating Serum-Zn did not alter ECF-Zn or BBB disruption, suggesting Serum-Zn was not a significant source of elevated ECF-Zn during cerebral ischemia.

### Neuronal-specific ZnT3 knockout reduces ECF-Zn and prevents ischemia-induced BBB damage

**Figure 3.**
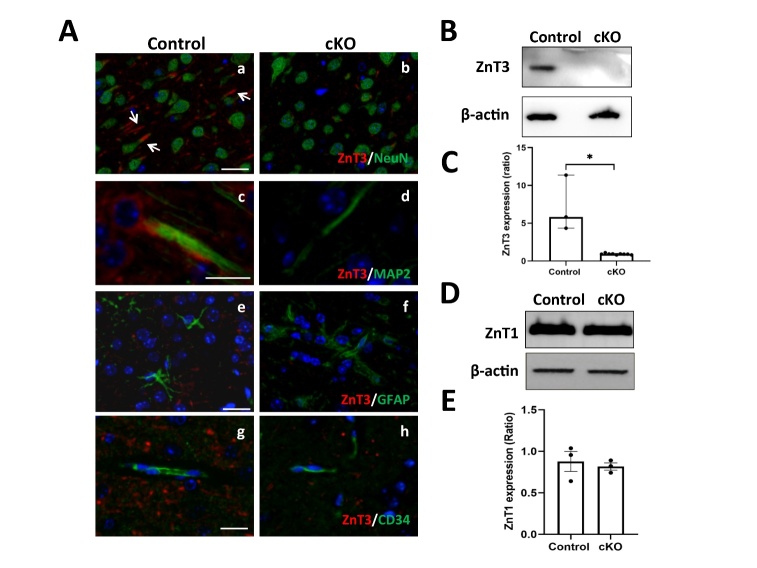
**ZnT3 expression in neuronal-specific conditional knockout mice. (A)** Histochemistry staining showing ZnT3 expression in brain tissues of control (fl/fl;+/+) or ZnT3 neuronal-specific conditional knockout mice (ZnT3-cKO, fl/fl; Syn1-icre/+). ZnT3 positive staining (red) in control mice **(a)** was mostly located around to the NeuN staining (a specific marker for neuronal nucleus, green), and extended to the axons-like structures (arrows). ZnT3 signals were also co-localized with microtubule-associated protein 2 (MAP2, green), a specific biomarker for axons and dendrites **(c)**. No positive ZnT3 signal was observed in the neurons of ZnT3-cKO mice **(b and d)**. ZnT3 signals in control or cKO mice were hardly observable either in GFAP-positive (an astrocyte-specific marker, green) or CD34-positive (endothelium specific markers, green) cells **(e-h)**. Bar: 20 μm. **(B/C)** Western blot analysis of ZnT3 expression in brain tissues of control (n=3) or ZnT3-cKO mice (n=9). (D/E) Western blot analysis ZnT1 expression (n=3 in each group). Data are presented as mean±SEM. *p<0.05.

**Figure 4.**
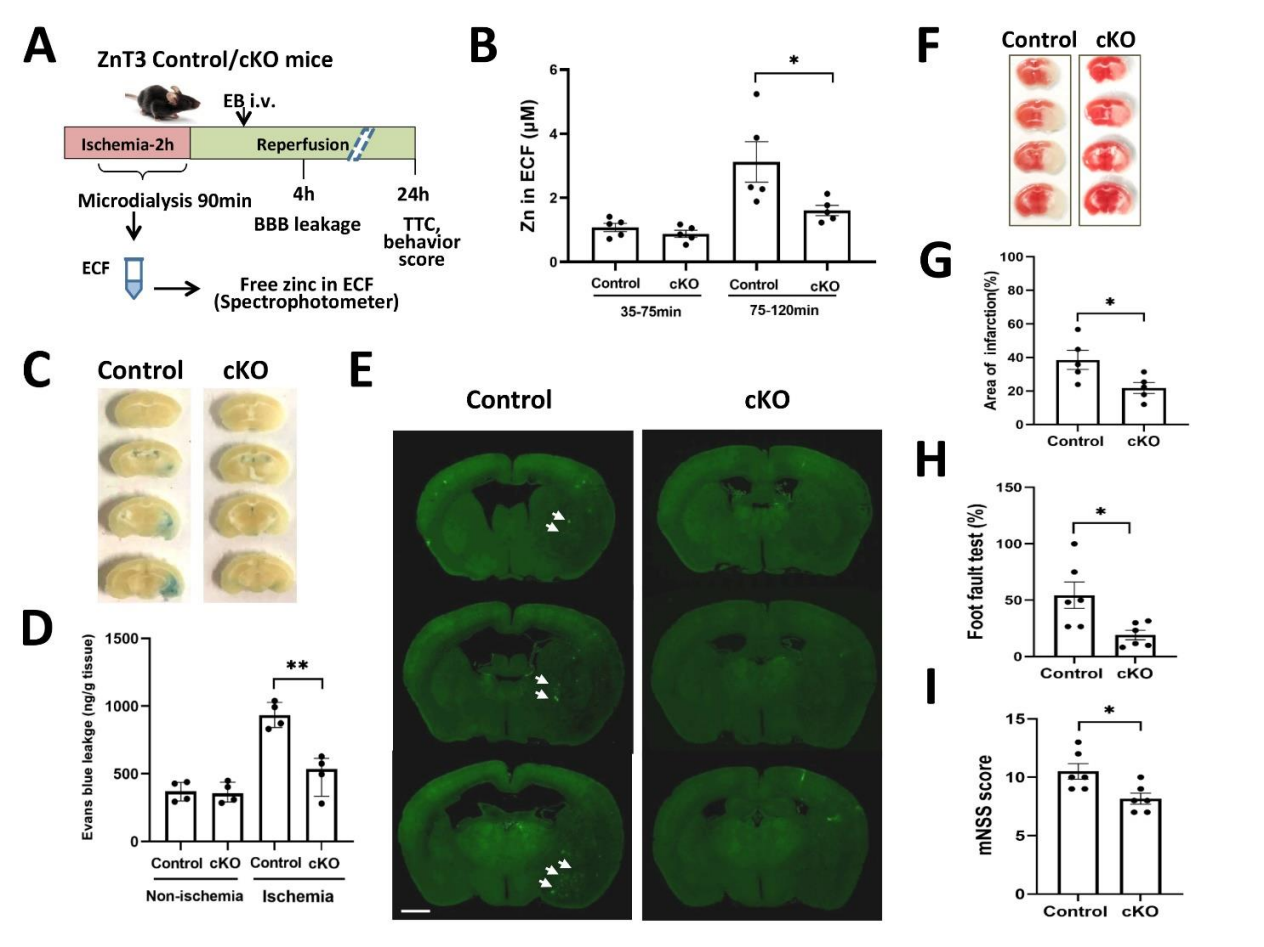
**Neuronal-specific ZnT3 knockout reduces ECF-Zn and prevents BBB damage after cerebral ischemia. (A)** The schematic diagram of experiments with control and ZnT3-cKO mice. We measured ECF-Zn level during ischemia and investigated ischemia-induced BBB disruption after 2h ischemia followed by 4h reperfusion. iv: intravenous injection. **(B)** ECF levels during early phase (30-75min) or later phase (75-120min) of ischemia. The level of ECF-Zn was measured spectrophotometrically (n=5 in each group). **(C/D)** Evan’s blue leakage in control and ZnT3-cKO mice, demonstrating that the ischemia-reperfusion caused BBB disruption, which was abolished by neuronal-specific ZnT3 knockout (n=4 in each group). **(E)** The endogenous IgG leakage after 2 h-ischemia/4h-reperfusion. Endogenous IgG extravasion (Arrows) was measured using immunohistochemistry staining. Scale bar: 1mm. **(F/G)** The measurement of cerebral infarction using TTC staining after 2h-ischemia/22h-reperfusion (n=5 in each group). **(H)** Neuronal function analysis using Foot fault test. (n=6 in each group) I. Neuronal function analysis using mNSS score. (n=6 in each group), *p<0.05, **p<0.01, Data are presented as mean±SEM (B-G) or median (interquartile range, H and I).

It is well recognized that zinc is highly enriched in synaptic vesicles and is released from neurons during cerebral ischemia. In order to investigate whether ECF-Zn comes from ischemic neurons and contributes to BBB disruption, we generated zinc transporter ZnT3 neuronal-specific conditional knockout (ZnT3-cKO) mice. ZnT3 expression in brain tissues of control or ZnT3-cKO mice was measured using histochemistry and Western blot to verify the efficacy of ZnT3 knockout in neurons. The histochemistry images showed that ZnT3 positive staining in control mice was mostly located around the NeuN (a specific marker for neuronal nucleus) staining and extended to the axons-like structures (arrows, Fig. 3Aa). ZnT3 signals were also co-localized with microtubule-associated protein 2 (MAP2), a specific biomarker for axons and dendrites (Fig. 3Ac). In contrast, no positive ZnT3 signal was observed in the neurons of ZnT3-cKO mice (Fig. 3A b and d). Moreover, ZnT3 signals in control or cKO mice (Fig. 3A e-h) were hardly observable either in GFAP-positive (an astrocyte-specific marker) or CD34-positive (endothelium specific markers) cells. Western blot analysis showed that a high level of ZnT3 expression was seen in brain tissues of control mice, while there was almost no ZnT3 expression in cKO mice (Fig. 3B/C). The expression of ZnT1, another zinc transporter in brain, remained unchanged (Fig. 3D/E). These results confirmed that generation of the mice with ZnT3 neuronal-specific conditional knockout was successful. Utilizing the ZnT3-cKO mice, we measured the level of ECF-Zn during 2h cerebral ischemia and investigated ischemia-induced BBB disruption at the end of 4h reperfusion. The schematic diagram is shown in Figure 4A.

There was no difference in the levels of ECF-Zn between control and cKO groups during the early phase of ischemia (30-75 min MCAO) (Fig. 4B). However, the ECF-Zn in control mice increased 3-fold during the later phase of ischemia (75-120 min MCAO), whereas ECF-Zn in ZnT3-cKO mice only had a marginal change, suggesting that neuronal-specific ZnT3 knockout prevented free zinc release from neurons during cerebral ischemia. BBB permeability demonstrated that ischemia/reperfusion significantly increased EB extravasation (Fig. 4C/D), and this increase was mostly eliminated in neuronal-specific ZnT3 knockout mice. We also measured the BBB damage using the endogenous IgG leakage from the compromised BBB (another widely accepted method beside the Evan’s blue extravasation). IgG leakage was significantly inhibited in ZnT3-cKO mice, compared with the control mice (Fig. 4E), consistent with the EB extravasation experiment (Fig. 4C/D).

To investigate the effects of ZnT3 knockout on infarction and neurological function at early phase of cerebral ischemia/reperfusion, we measured the cerebral infarction and neurological function in ZnT3-cKO and control mice after ischemia/reperfusion at 24h. The results show that neuronal ZnT3 knockout significantly reduced cerebral infarction (TTC staining, Fig. 4F/G) and improved the neuronal functions (Foot fault test: Fig. 4H and mNSS score: Fig. 4I).

These findings suggest that the elevated level of ECF-Zn during cerebral ischemia came from neurons via the action of ZnT3, which contributed to BBB disruption.

### ECF-Zn directly binds and activates MMP-2 in extracellular fluid

We next investigated potential mechanism(s) by which elevated levels of ECF-Zn could mediate ischemia-caused BBB damage. As an important zinc-containing proteinase, MMP-2/-9 is crucial for degrading tight junction proteins, thus compromising BBB integrity during cerebral ischemia. We hypothesized that ischemia-released ECF-Zn may directly activate MMP-2/-9 in extracellular fluid (ECF-MMP-2/-9).

**Figure 5.**
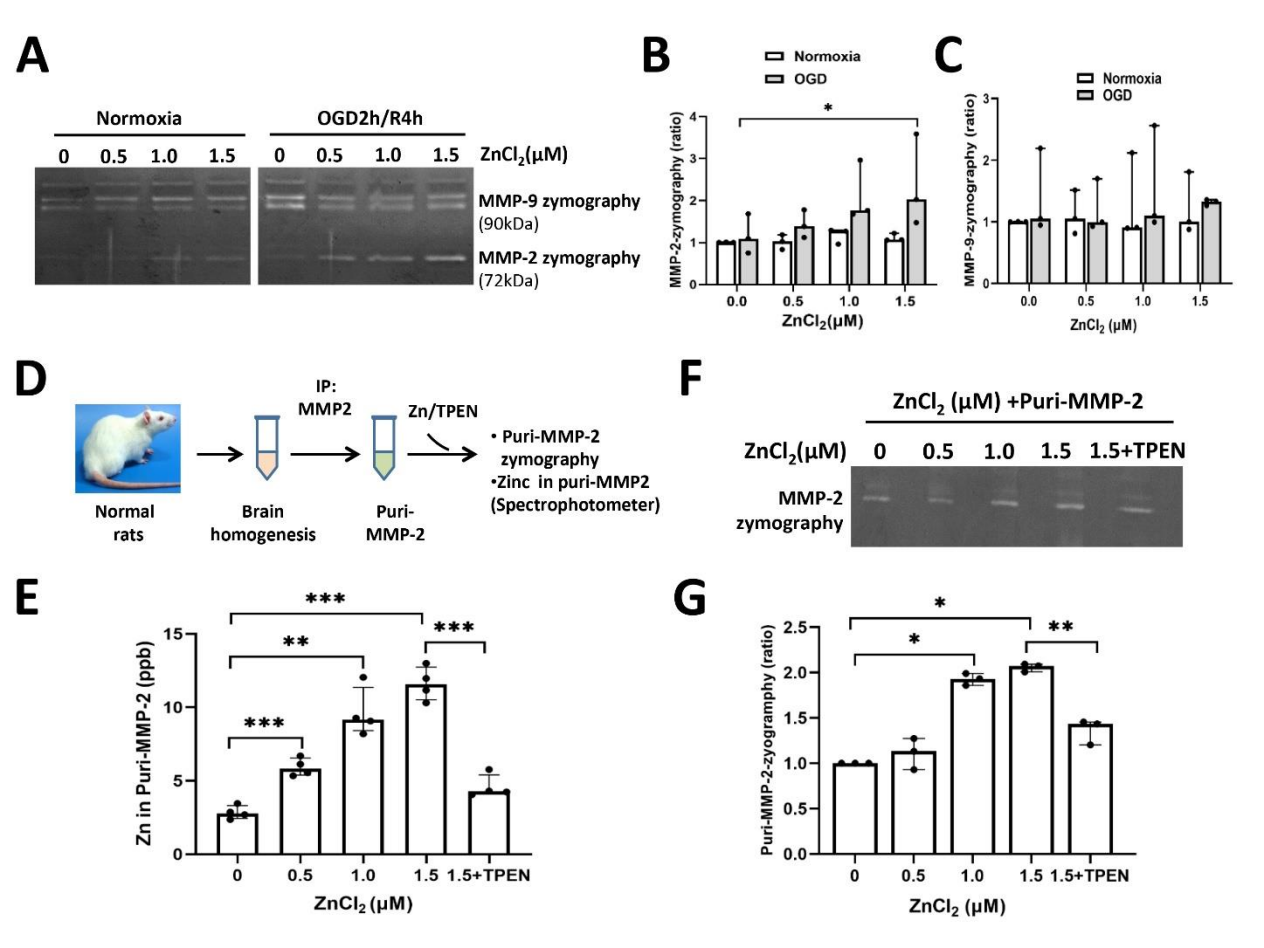
**ECF-Zn directly binds and activates MMP-2 in extracellular fluid. (A)** Varying concentrations of ZnCl_2_ were added to OGD-treated endothelial cells, and conditional culture medium was collected after 120min OGD/4h reperfusion and purified to measure the activity of MMP-2/-9 by gelatin zymography. **(B/C)** Quantitative analysis of extracellular MMP-2/-9 activity in cultured medium after OGD/Reperfusion (Data are presented as mean±SEM, n=3, three separate independent experiments). *p<0.05. **(D)** The schematic diagram of establishing an *in vitro* system to mimic the extracellular environment. Varying concentrations of ZnCl_2_ or zinc chelator, TPEN was incubated with purified MMP-2, which was isolated from the brain homogenate of naïve rats. **(E)** Spectrophotometric measurement of the level of zinc that binds to MMP-2 protein. The zinc content in MMP-2 from brain tissue was relatively low, but increased concentration dependently with added zinc in the incubation media. Zinc chelator TPEN could remove MMP-2 protein-bound zinc (n=4). ** p<0.01, *** p<0.001. **(F)** Gelatin zymography showing the activity of purified-MMP-2 in response to different concentration of zinc in surroundings. **(G)** Quantitative analysis showing that purified-MMP-2 was gradually activated in accordance with the elevation of free zinc concentration in the surroundings (n=3). *p<0.05, **p<0.01. Data are presented as mean±SEM.

To mimic the elevated level of ECF-Zn, ZnCl_2_ at varying concentrations was added to oxygen/glucose deprivation (OGD)-treated endothelial cells, and the resulting culture medium was collected after 120min OGD/4h reperfusion for evaluating MMP-2/-9 activity by gelatin zymography. Extracellular MMP-2 (Fig. 5A/B), but not MMP-9 (Fig. 5A/C), in ECs cultured medium was significantly activated in a zinc-concentration dependent manner, suggesting that free zinc participated in regulating MMP-2 activity in the extracellular medium.

To further study the mechanism of ECF-Zn in regulating ECF-MMP-2 activity in cultured medium, we established an *in vitro* system to mimic the extracellular environment. We first isolated MMP-2 by immunoprecipitation from the brain homogenates of naïve rats, and then incubated the purified MMP-2 with varying concentrations of ZnCl_2_ or the zinc chelator, TPEN. The MMP-2 protein was then collected for analysis of its Zn content and enzymatic activity, as shown in the schematic diagram (Fig. 5D). The level of MMP-2 bound zinc was relatively low in purified MMP-2 isolated from rat brain tissue. Intriguingly, MMP-2 bound zinc increased significantly with increasing concentration of added zinc in the incubation solution. Moreover, the zinc chelator TPEN could sequester MMP-2 bound zinc (Fig. 5E). The activity of purified MMP-2 was also evaluated after incubation with different concentration of zinc using gelatin zymography (Fig. 5F/G). The quantitative analysis showed that MMP-2 activity gradually increased in accordance with the elevation of free zinc concentration in the surroundings. These results suggest a novel mechanism of MMP-2 activation through elevated levels of free zinc directly binding to MMP-2 and increasing its activity.

**Figure 6.**
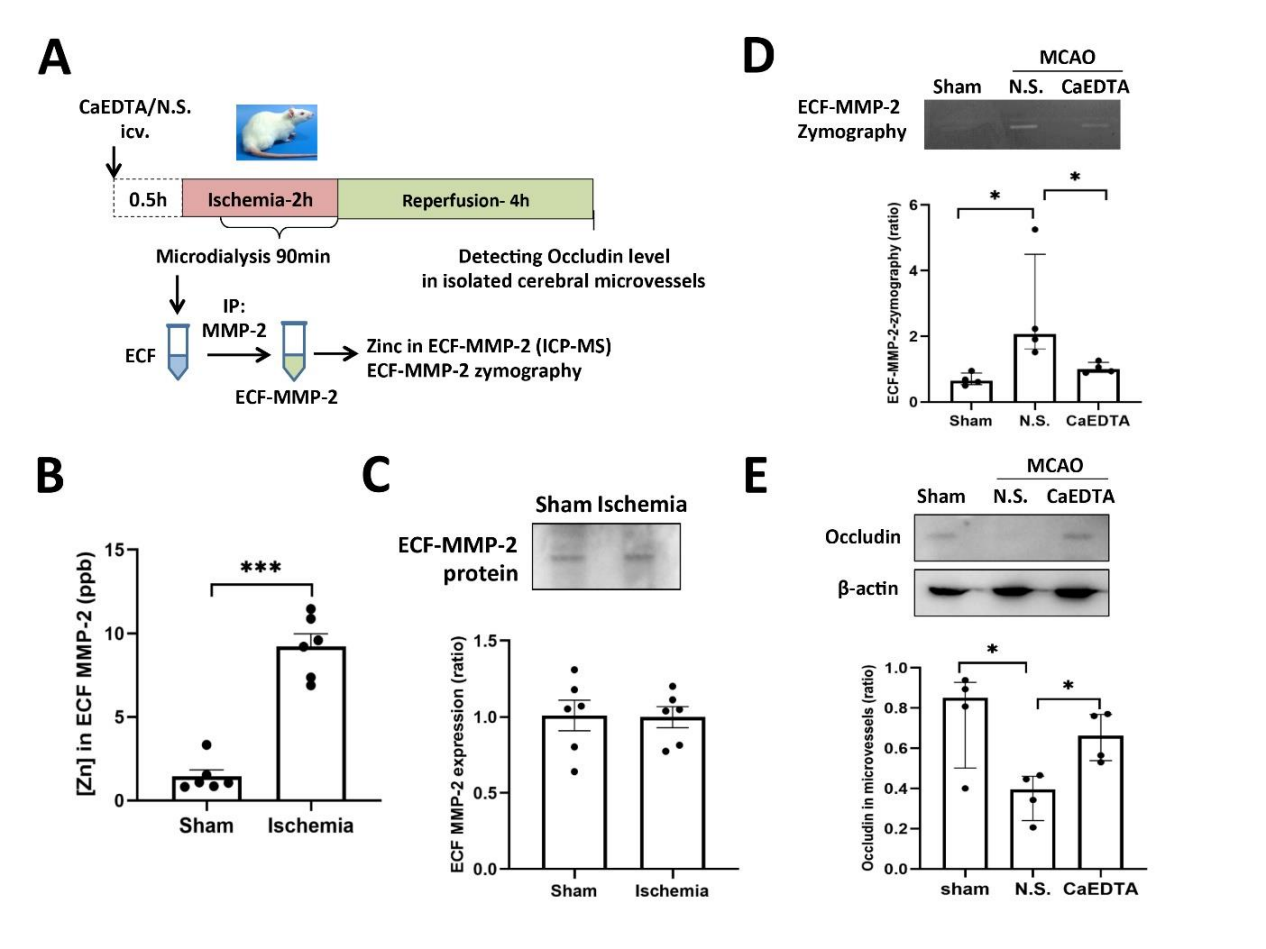
**ECF-Zn directly binds and activates ECF-MMP-2 in ischemic rats, leading to the loss of occludin from cerebral microvessels during ischemia. (A)** The experimental schematic diagram investigating the role of ECF-Zn in activating ECF-MMP-2 in the brain of ischemic rats. CaEDTA (a membrane-impermeable zinc chelator) or saline (N.S.) was microinjected in lateral ventricle 30min prior to ischemia to remove ECF-Zn in the brain. IP: immunoprecipitation; ICP-MS: inductively coupled plasma-mass spectrometry. **(B)** ECF was collected during cerebral ischemia to obtain purified ECF-MMP-2 protein. The level of zinc that is bound to ECF-MMP-2 was determined by ICP-MS. (n=6 in each group). ***p<0.001. **(C)** The level of ECF-MMP-2 protein by Western blot (n=6 in each group). **(D)** Analysis of ECF-MMP-2 activation using gelatin zymography (n=4 in each group). *p<0.05. **(E)** Western blot showing the level of occludin specifically in microvessels isolated from the ischemic hemisphere of rats. Quantitative analysis of Western blot showed that ischemia/reperfusion induced occludin loss, while removing ECF-Zn by CaEDTA microinjection rescued the loss of occludin in microvessels (n=4 in each group). *p<0.05. Data are presented as mean±SEM.

### ECF-Zn directly binds and activates ECF-MMP-2 in ischemic rats, leading to the loss of occludin from cerebral microvessels during ischemia

We further investigated the role of ECF-Zn in activating ECF-MMP-2 in the brains of ischemic rats. The experimental schematic diagram is shown in Figure 6A. The ECF of rats was collected during cerebral ischemia and ECF-MMP-2 was immunoprecipitated to obtain purified protein. The level of zinc that bound to ECF-MMP-2 was then determined using inductively coupled plasma-mass spectrometry (ICP-MS). The level of zinc in ECF-MMP-2 was dramatically elevated during cerebral ischemia (Fig. 6B), while the western blot data demonstrated that the level of ECF-MMP-2 protein remained constant (Fig. 6C). In order to investigate the role of ECF-Zn in regulating the activity of ECF-MMP-2, the membrane-impermeable zinc chelator CaEDTA was microinjected in the lateral ventricle 30min prior to ischemia to remove ECF-Zn that would be generated in the brain during ischemia. The activity of ECF-MMP-2 was elevated during cerebral ischemia, but the increased activity was mostly abolished when ECF-Zn was removed with CaEDTA (Fig. 6D).

Occludin, an integral tight junction protein in microvessels, is a substrate of MMP-2. MMP-2 mediated occludin degradation leads to BBB disruption during cerebral ischemia. Here we examined whether ischemia-induced occludin loss in microvessel tissues was caused by elevated ECF-Zn levels. To specifically evaluate the level of occludin in cerebral microvessels, we extracted the microvessels from the ischemic hemisphere of rat with or without CaEDTA treatment. Western blot data showed that ischemia/reperfusion induced severe occludin loss in the isolated microvessels when compared with sham group (Fig. 6E). Most importantly, removing ischemia-elevated ECF-Zn by CaEDTA microinjection rescued the loss of occludin in microvessels. These findings reveal a novel mechanism by which ECF-Zn binds directly with ECF-MMP-2 to increase its activity, thereby degrading occludin in microvessels.

**Figure 7.**
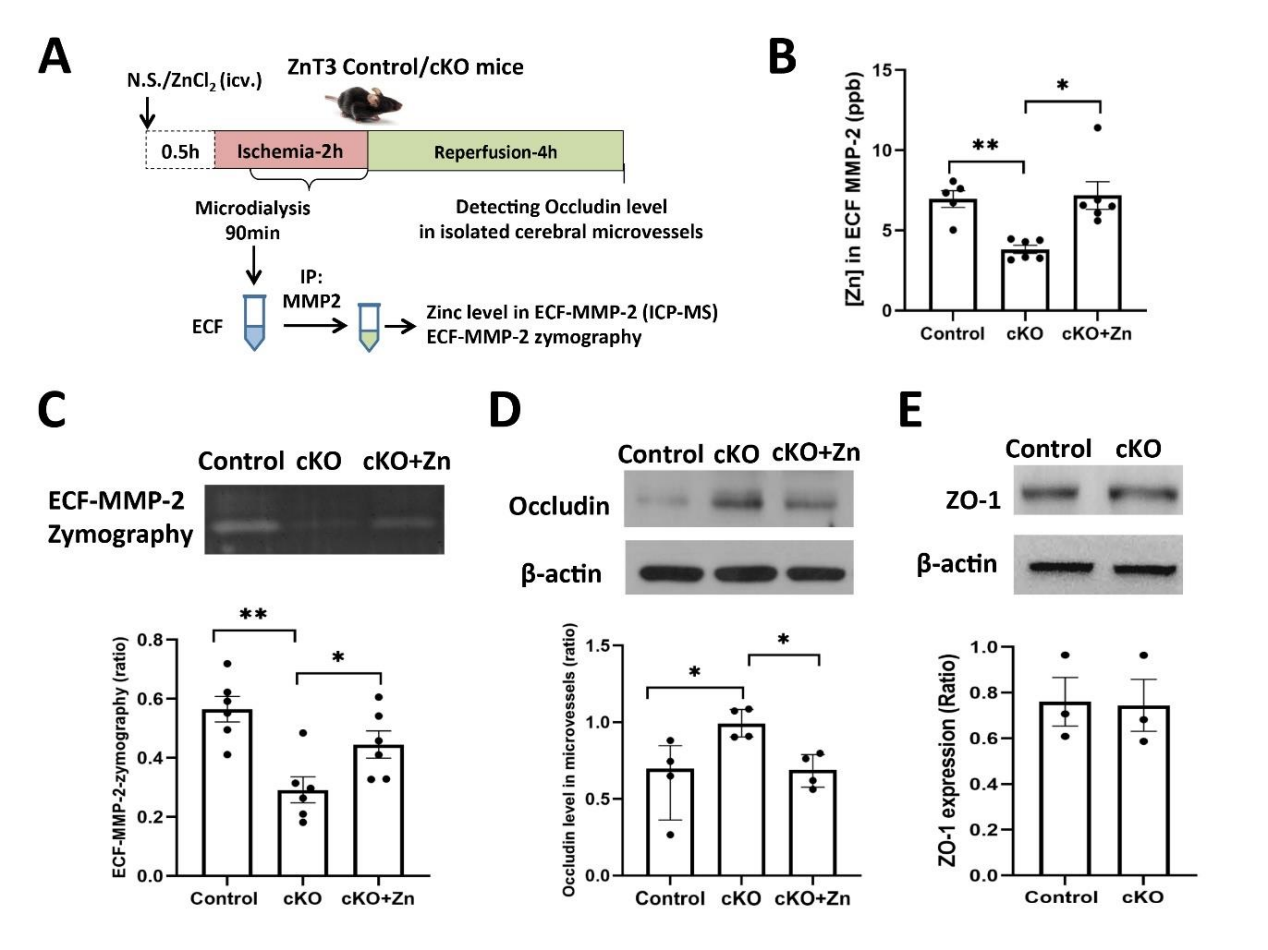
**Neuronal-specific ZnT3 knockout suppresses ECF-MMP-2 activation and prevents the loss of occludin from cerebral microvessels after ischemia. (A)** The schematic diagram of experiments to investigate the role of ECF-Zn in regulating ECF-MMP-2 activity and occludin loss in ZnT3-cKO (fl/fl; Syn1-icre/+) and control (fl/fl;+/+) mice after 2h ischemia followed by 4h reperfusion. ZnCl_2_ or saline was microinjected into lateral ventricle 30min before onset of MCAO to increase the level of ECF-Zn. The ECF was collected during cerebral ischemia to obtain purified ECF-MMP-2 protein. icv: lateral ventricle microinjection; IP: immunoprecipitation; ICP-MS: inductively coupled plasma-mass spectrometry. **(B)** ICP-MS data showed that the level of zinc bound to ECF-MMP-2 in control mice was elevated during cerebral ischemia, while neuronal-specific ZnT3 knockout reduced the level of zinc in ECF-MMP-2. ZnCl_2_ microinjection into lateral ventricle reversed the effect of ZnT3 knockout on zinc bound to ECF-MMP-2 (control: n=5; cKO: n=6; cKO+Zn: n=6). *p<0.05, **p<0.01. **(C)** Analysis of ECF-MMP-2 activation using gelatin zymography. ECF-MMP-2 activity was greatly reduced in ZnT3-cKO mice when compared with control mice, and ZnCl_2_ administration in lateral ventricle reversed the reduction of ECF-MMP-2 activity in ZnT3-cKO mice. (n=6 in each group) * p<0.05, ** p<0.01. **(D)** Western blot of occludin in isolated microvessels from ischemic brain. The loss of occludin from microvessels was significantly prevented in ZnT3-cKO mice when compared with control mice. ZnCl_2_ microinjection into lateral ventricle abolished the protective effects on occludin loss in ZnT3-cKO mice (n=4 in each group). **(E)** Western blot of ZO-1 expression in isolated microvessels after 2h-ischemia/4h-reperfusion (n=3 in each group). Data are presented as mean±SEM. * p<0.05.

### Neuronal-specific ZnT3 knockout suppresses ECF-MMP-2 activation and prevents the loss of occludin from cerebral microvessels after ischemia

We further investigated the role of ECF-Zn on regulating ECF-MMP-2 activity and occludin loss by utilizing ZnT3-cKO mice with 2h ischemia followed by 4h reperfusion. ZnCl_2_ or saline was microinjected into lateral ventricles 30 min before the onset of MCAO to alter the level of ECF-Zn. The ECF of mice was collected to obtain immuno-purified ECF-MMP-2 protein for measuring its activity and zinc content. The schematic diagram is shown in Figure 7A. As expected, neuronal-specific ZnT3 knockout significantly reduced the level of zinc in ECF-MMP-2 protein. In contrast, ZnCl_2_ administration into extracellular surroundings by microinjection into the lateral ventricle recapitulated the increase in ECF-MMP-2 seen in control mice (Fig. 7B). Accordingly, the activity of ECF-MMP-2 was greatly reduced in ZnT3-cKO mice when compared with control mice, and ZnCl_2_ administration in lateral ventricle mostly reversed the reduction of ECF-MMP-2 activity in ZnT3-cKO mice (Fig. 7C). Finally, Western blot of microvessels isolated from the ischemic brain showed that neuronal-specific ZnT3 knockout prevented the loss of occludin, while ZnCl_2_ microinjection into the lateral ventricle abolished the protective effects on occludin loss observed in ZnT3-cKO mice (Fig. 7D). There was no significant change in ZO-1 expression (a tight junction protein modulated by MMP-9) between control and cKO mice in response to 2h-ischemia/4h-reperfusion (Fig. 7E), indicating the critical role of MMP-2/occludin pathway in neuronal ZnT3-modulaed BBB disruption in response to cerebral ischemia. These results suggest that ischemia-induced elevation of ECF-Zn plays a critical role in regulating ECF-MMP-2 activity and in promoting occludin loss from microvessels in ischemic brain. The recapitulation of all wild-type phenotypes missing in ZnT3-cKO mice by simply adding Zn to increase ECF-Zn demonstrates the central importance of this process to BBB damage.

## DISCUSSION

BBB disruption in the acute phase of ischemic stroke has been associated with hemorrhagic transformation, inflammation, and edema after recanalization, contributing significantly to the overall brain injury in stroke patients [30]. BBB integrity is maintained by the neurovascular units, which are composed of neurons, perivascular astrocytes, microglia, pericytes, endothelial cells, and the basement membrane [31]. The components of the neurovascular unit share intimate and complex associations, and collectively act as a single functioning unit. It is increasingly recognized that the connections and interactions within the neurovascular unit play integrative roles in response to ischemic insults [32]. Therefore, it is fundamental to elucidate the aberrant signaling in the neurovascular unit that is generated in response to ischemia and understand how the aberrant signaling triggers the cascade of molecular events leading to the disruption of BBB and brain injury following stroke.

In the present study, we report that elevated levels of ECF-Zn, which arises from ischemic neurons, serve as an aberrant signal causing ischemia-induced BBB damage in ischemic stroke rats. Specific neuronal knockout of ZnT3 blocked free zinc release from neurons to extracellular surroundings and reduced the levels of ECF-Zn, preventing BBB disruption during cerebral ischemia. Our findings reveal that aberrant signaling by the ischemic neurons themselves plays a critical role in the early phase of BBB damage during cerebral ischemia, suggesting that targeting this neuronal aberrant signaling might prove to be an effective strategy for attenuating ischemia-mediated vascular injury.

Neurons are highly sensitive to ischemia due to their high demands for oxygen and nutrition [33]. Zinc, which is abundant in neurons, plays critical roles in neuronal activities under both physiological and pathological conditions [7]. Our recent studies demonstrate that zinc accumulated in microvessels and contributed to BBB disruption in the acute phase of cerebral ischemia [5]. The present study investigated the mechanism of zinc transport from neurons to their extracellular surroundings, providing direct evidence that zinc released from ischemic neurons may serve as an aberrant extracellular signal leading to BBB damage. Our data showed that the elevated level of ECF-Zn during ischemia mainly came from the release of zinc from synaptic vesicles of ischemic neurons, as specific neuronal knockout of ZnT3 significantly suppressed the level of ECF-Zn in extracellular surroundings and prevented the ischemia-induced BBB disruption. These findings suggest that ZnT3 might be a promising target for developing novel drugs to avert ischemic-induced BBB damage through its inhibition.

Recent studies showed that rapid endothelial cytoskeletal reorganization enables early BBB disruption and the ischemic/reperfusion brain injury, suggesting that BBB disruption may be a cause rather than a consequence of parenchymal cell injury [34]. The present study reported that the neuronal-specific ZnT3 knockout mice not only reduced ECF-Zn and BBB permeability, but also suppressed cerebral infarction after ischemia, demonstrating that ZnT3 as an effective target for BBB protection. The mechanisms underlying need to further investigate. Studies showed that, in pathological conditions, zinc may be released from presynaptic neurons and astrocytes and resulted in cell death via upregulating oxidative stress. Therefore, we inferred that high level of extracellular fluid (ECF-Zn) is probably to lead to pan-necrosis after cerebral ischemia. Further investigations are underway to better understand this novel mechanism.

We further studied how elevated level of ECF-Zn induced BBB disruption during cerebral ischemia. MMP-2 and MMP-9 are members of a large family of endopeptidases, containing a zinc-binding site at the catalytic domain. The mechanism of “cysteine-zinc switch” has been generally accepted for regulating the MMP-2/-9 zymogen that the conserved cysteine at carboxyl terminus acts as a fourth inactivating ligand for the catalytic zinc atom in the active site to render the enzyme inactive [14]. For the enzyme to be activated (such as by oxidants), this cysteine-zinc pairing is disrupted, and the active site is exposed to cleave the peptide bonds of its substrates. However, this traditional activation mechanism could not explain the relationship between the level of MMP-2 bound zinc and its activation observed in the present study. Our findings reveal a novel mechanism in MMP-2 activation, besides the traditional “cysteine-zinc switch” model. We propose that zinc is an extracellular signaling molecule regulating MMP-2 activity. Under physiological conditions, levels of ECF zinc are low and the zinc-binding site in the catalytic domain might be vacant and inactive. In contrast, cerebral ischemia greatly elevated the concentration of ECF-Zn, which then binds to ECF-MMP-2 and activates the enzyme, leading to ischemia-induced occludin degradation and BBB disruption. Importantly, the increased levels of ECF-zinc observed *in vivo* were sufficient to cause MMP-2 activation in our *in vitro* system, demonstrating that this process occurs at relevant levels of ischemia-increased ECF zinc. Intriguingly, activation of MMP-2, but not MMP-9, by elevated free zinc was observed in our study. The reasons for the differential activation effect are not yet clear. Further investigations on zinc binding to MMPs and their activation are underway to better understand this novel mechanism and differential MMP activation.

**Limitations:** 1) Studies showed that gender has impacts on the prognosis of ischemic stroke. In the present study, male animals were used to increase the homogeneity and test our mechanistic hypothesis. Future studies will investigate the potential difference between two sexes. 2) The present study is a mechanistic investigation (not a therapeutic study), and the goal is to test a novel hypothesis about the source of high level of zinc in extracellular fluid (ECF-Zn) and the mechanism by which ECF-Zn mediates ischemia-induced BBB damage. Therefore, in order to minimize potential compounding factors complicating the mechanisms, we chose to use healthy and young animals in our designed experiments. Future study will further investigate the current mechanism in animal models with increased stroke risk factors, such as age, sexes, hypertension, diabetes.

## Conclusion

The present study shed new light on our understanding of the mechanisms of ischemia-induced BBB disruption. Moreover, ZnT3 appears crucial for regulating zinc transportation from neuronal synaptic vesicles to their extracellular surroundings and could be a potential target for developing novel drugs to prevent ischemic-induced BBB damage.
